# Trimethylamine *N*-Oxide, a Bariatric Surgery-Associated Host-Microbial Cometabolite, Reduces Colonic Tumorigenesis in a Sex-Dependent Manner

**DOI:** 10.1016/j.jcmgh.2026.101869

**Published:** 2026-08-29

**Authors:** Yang Bi, Maria Glymenaki, Maria A. Valdivia-Garcia, Mu Wang, Kai Yee Eng, Edward Lin, Nana Gletsu-Miller, Julian R. Marchesi, Ron Smits, Mona El-Bahrawy, Nadia Guerra, Jia V. Li

**Affiliations:** 1 Section of Nutrition, Department of Metabolism, Digestion and Reproduction, Faculty of Medicine, Imperial College London, London, United Kingdom; 2 European Food Safety Authority, Parma, Italy; 3 Department of Surgery, Emory University, Atlanta, Georgia; 4 Department of Applied Health Science, School of Public Health, Indiana University Bloomington, Bloomington, Indiana; 5 Department of Gastroenterology and Hepatology, Erasmus MC Cancer Institute, University Medical Center Rotterdam, Rotterdam, The Netherlands; 6 Faculty of Natural Sciences, Department of Life Science, Imperial College London, London, United Kingdom

**Keywords:** Bacterial Metabolism, Colon Cancer Risk, Cytokines, DNA Damage, Inflammation, Metabolism, Microbiota, Weight Loss Surgery

## Abstract

**Background & Aims:**

Trimethylamine *N*-oxide is a host–microbial cometabolite that significantly increases following Roux-en-Y gastric bypass. Although trimethylamine *N*-oxide is associated with cardiovascular diseases, its role in colorectal cancer remains unclear.

**Methods:**

Fabpl*Cre;Apc*^*15lox/+*^ mice, a genetically altered colorectal cancer model, together with murine macrophages and human colonic cancer cells (HCT-116), were used to investigate the impact of trimethylamine *N*-oxide on colonic tumorigenesis.

**Results:**

Trimethylamine *N*-oxide supplementation significantly reduced the colonic tumor load in male, but not female, mice. Consistently, dietary trimethylamine *N*-oxide resulted in higher retention of circulating trimethylamine *N*-oxide in males, which was significantly and inversely correlated with tumor loads. Furthermore, tumor necrosis factor-α–expressing cell frequencies in the colonic intraepithelial lymphocytes were significantly lower in trimethylamine *N*-oxide–supplemented male mice compared with controls, suggesting that circulating trimethylamine *N*-oxide could play a protective effect against colonic tumor growth via down-regulation of tumor necrosis factor-α–expressing cells. This was supported by in vitro observations that trimethylamine *N*-oxide reduced lipopolysaccharide-stimulated tumor necrosis factor-α production from macrophages. Trimethylamine *N*-oxide exerted no effects on cell proliferation (Ki67) and DNA damage (γH2AX) of HCT-116 cells.

**Conclusions:**

Our study leads us to conclude that higher retention of circulating trimethylamine *N*-oxide has a protective effect against colorectal cancer in a sex-dependent manner, highlighting the importance of understanding the complex relationship among the concentrations of host–microbial cometabolite trimethylamine *N*-oxide, its systemic circulation, and its biological function in modulating colorectal cancer risk.

## Graphical abstract

**Figure:**
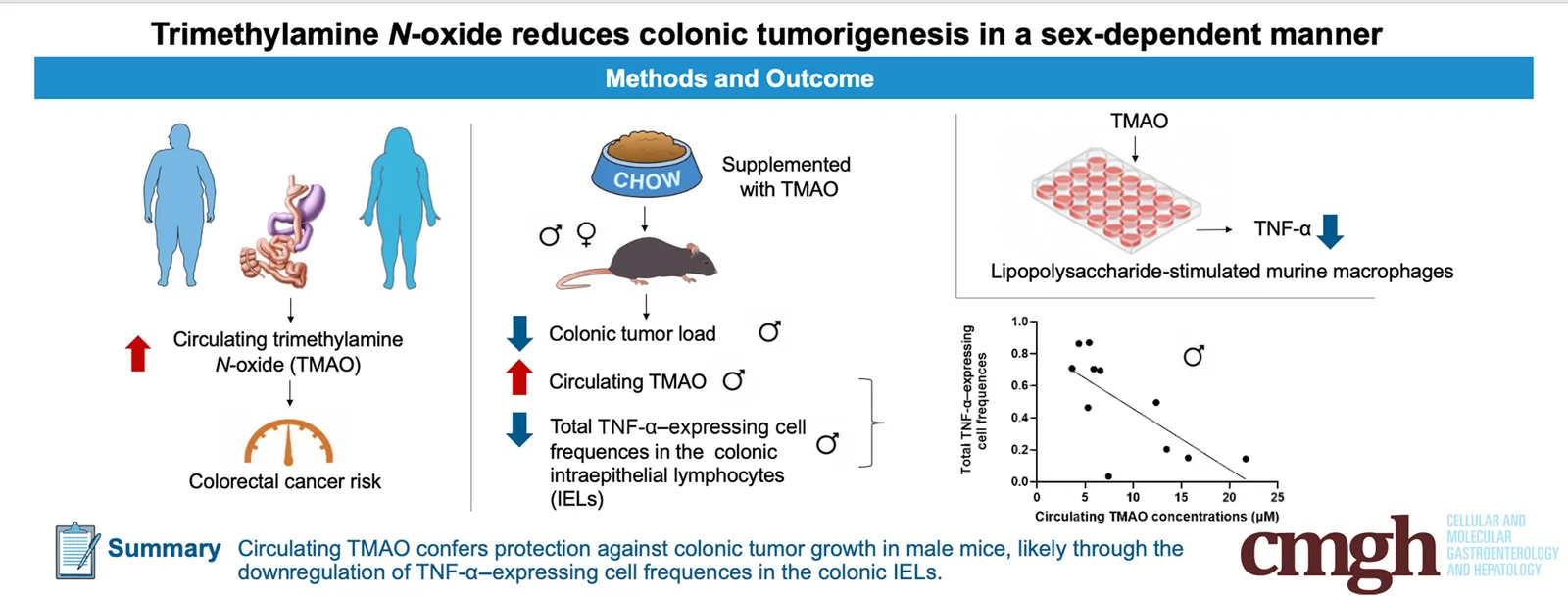

**Summary**

Although the association between bariatric surgery and colorectal cancer risk remains controversial, Roux-en-Y gastric bypass has consistently been shown to elevate circulating trimethylamine *N*-oxide levels, which have been associated with higher colorectal cancer risk. However, our findings reveal that dietary trimethylamine *N*-oxide supplementation results in greater systemic retention of trimethylamine *N*-oxide in male compared with female mice. This higher circulating trimethylamine *N*-oxide level confers protection against colonic tumor growth in a sex-dependent manner, likely through the downregulation of tumor necrosis factor-α–expressing cell frequencies in colonic intraepithelial lymphocytes.

**What You Need to Know**

**Background**

Trimethylamine *N*-oxide increases markedly after Roux-en-Y gastric bypass. Roux-en-Y gastric bypass has been associated with an elevated risk of colorectal cancer, although evidence remains mixed.

**Impact**

Dietary trimethylamine *N*-oxide reduced colonic tumor burden and increased systemic trimethylamine *N*-oxide retention in male mice, suggesting a sex-dependent protective effect potentially mediated by reduced tumor necrosis factor-α–expressing colonic intraepithelial lymphocytes.

**Future Directions**

Elucidating trimethylamine *N*-oxide–mediated modulation of tumor necrosis factor-α signaling in human populations, together with mechanisms underlying sex-specific trimethylamine *N*-oxide retention, is essential for informing targeted dietary interventions aimed at reducing colorectal cancer risk.

Bariatric surgery, including Roux-en-Y gastric bypass (RYGB) and sleeve gastrectomy, induces sustained weight loss and improves obesity-related comorbidities.^1^ However, several cohort studies highlighted the potential association between bariatric surgery and increased colorectal cancer (CRC) risk.^2^^,^^3^ Furthermore, RYGB is associated with a significant shift in the gut microbiota towards enrichment of members from the *Enterobacteriaceae* family and increased host–microbial cometabolites, such as trimethylamine *N*-oxide (TMAO) in biofluids.^4^, ^5^, ^6^, ^7^ TMAO is produced from oxidation of trimethylamine (TMA) by flavin-containing monooxygenase 3 in the liver.^8^ TMA is mainly a bacterial metabolite produced from dietary choline and carnitine, catalyzed by enzymes, such as choline-TMA lyase and carnitine monooxygenase.^9^ The higher abundance of *cutC* genes has been reported in nonbariatric patients with CRC.^10^^,^^11^

TMAO has been known historically as an osmolyte, and it stabilizes the structural and functional integrity of proteins.^12^ However, there has been much interest in the role of TMAO with respect to inflammation in cardiovascular disease, chronic kidney disease, and CRC.^13^, ^14^, ^15^, ^16^ A genome-wide analysis showed that TMAO was associated with CRC,^17^ and a positive correlation between plasma TMAO levels and CRC was found in the Women’s Health Initiative Observational Study.^18^ In a more recent study, higher serum TMAO levels were found to be associated with a higher risk of distal CRC, but not overall CRC risk.^19^ The empirical mechanistic evidence on the function of TMAO in CRC remains sparse. A rodent-based study showed that TMAO exerted an oncogenic effect by enhancing cell proliferation and angiogenesis in CRC.^20^ In contrast, findings on the protective function of TMAO have also been reported. Patients with triple-negative breast cancer showed better responses to immunotherapy with higher circulating TMAO concentrations, and TMAO levels were higher in tumors with an activated immune microenvironment.^21^ Furthermore, intraperitoneal delivery of TMAO or dietary choline supplementation reduced tumor growth in an orthotopic pancreatic ductal adenocarcinoma mouse model and was associated with an immunostimulatory tumor–associated macrophage phenotype and an activated effector T cell response in the tumor microenvironment.^22^ These findings collectively suggested that TMAO potentially regulates immune function and tumor development.

To investigate the causal relationship between TMAO and CRC risk, we employed a Fabpl*Cre*;*Apc*^15lox/+^ mouse model and combined methodologies including flow cytometry, histology, and metabolomics. Fabpl*Cre*;*Apc*^15lox/+^ mice better mimic the early-stage development of human sporadic CRC with tumors primarily developed in the large intestine compared with *Apc*^min/+^ mice.^23^ Based on the findings from the mouse model, we further explored the impact of TMAO on CRC risk factors, including cell proliferation, DNA damage, and inflammation, using macrophages and colon cancer cells.

## Results

### Dietary Supplementation With Trimethylamine *N*-Oxide Significantly Reduces Colonic Tumorigenesis in Male Mice

Prior to investigating the effects of TMAO in a mouse model, we first quantified circulating levels of TMAO and TMA in patients undergoing bariatric surgery. Bariatric surgery procedures primarily include restrictive techniques, such as laparoscopic gastric banding (LGB), reducing food intake, and the combined restrictive and malabsorptive methods, such as RYGB, limiting both food consumption and nutrient absorption.^24^ Circulating and urinary TMAO have been found to be elevated following RYGB surgery, in particular, patients with type 2 diabetes mellitus (T2DM).^5^^,^^7^^,^^24^ In our cohort of 16 patients (RYGB, n = 11; LGB, n = 5) (Table 1), we quantified circulating TMAO and TMA using ultra-performance liquid chromatography-tandem mass spectrometry (UPLC-MS/MS)^25^ and observed consistent findings of increased concentrations of TMAO in fasting serum samples at 6 months post-RYGB compared with preoperatively (*P* = .002) (Figure 1*A*), but this elevation was not noted in the LGB group. Furthermore, TMAO concentrations were significantly higher in the RYGB group compared with the LGB group at 6 months (*P* = .0496) (Figure 1*A*). In contrast, circulating concentrations of TMA did not exhibit statistically significant changes across time points nor between the 2 surgical groups (Figure 1*B* and *D*). Additionally, the increases in TMAO levels following RYGB were observed in groups with and without T2DM (Figure 1*C*).

**Table 1:** Demographic and Clinical Characteristics of Patients who Underwent Bariatric Surgery at Baseline (Preoperative) and 6 Months Postoperatively (6 Mo)

	LGB			RYGB		
	Preoperative	6 Mo	*P*	Preoperative	6 Mo	*P*
No. patients (glycemia)	5 (1)			11 (6)		
Race (Black:White)	4:1			4:7		
Age, *y*	31 ± 9.09			41 ± 8.21		
VAT, *cm*^*3*^	2696 ± 1859.95	2272 ± 1319.84	ns	6022 ± 2209.08	2096 ± 1378.57	.001
SAT, *cm*^*3*^	12,674 ± 2557.7	10,806 ± 3427.74	ns	16,601 ± 4408.35	9564 ± 3258.91	.001
CRP, *mg/dL*	0.79 ± 0.33	0.2 ± 0.43	ns	1.28 ± 1.15	0.41 ± 0.48	.004
FAT, *kg*	53.3 ± 14.29	43.5 ± 15.21	ns	70 ± 12.87	40.2 ± 13.41	.001
WC, *cm*	114.2 ± 11.28	99.2 ± 10.47	ns	126.8 ± 14.36	102 ± 13.49	.001
GLC, *mM*	4.28 ± 0.48	4.83 ± 1.76	ns	6.06 ± 1.75	4.2 ± 0.59	.001
INS, *uIU/mL*	15.6 ± 18.58	6.5 ± 3.22	ns	11.4 ± 9.37	3.1 ± 1.41	.001
HOMA	2.84 ± 4.6	1.61 ± 0.52	ns	2.63 ± 4.28	0.57 ± 0.33	.001
BMI, *kg/m*^*2*^	39.59 ± 5.72	34.86 ± 6.43	ns	45.27 ± 3.43	34.26 ± 4.79	.001
SBP, *mm Hg*	115.5 ± 15.78	128 ± 10.52	ns	137 ± 17.42	119.4 ± 16.33	.011
DBP, *mm Hg*	65 ± 10.21	76.7 ± 9.74	ns	72.3 ± 11.88	71 ± 11.06	ns
REE, *kcal*	2327 ± 241.64	1720 ± 260.52	ns	2051 ± 415.81	1647 ± 284.13	.002

NOTE. Data are presented as number or median ± standard deviation.

*P* values derived from Wilcoxon matched-pairs signed rank test (2-tailed); ns, non-statistically significant (*P* > .05).

BMI, body mass index; CRP, high sensitivity C-reactive protein; DBP, diastolic blood pressure; FAT, body fat mass by air displacement plethysmography; GLC, fasting glucose; HOMA, homeostasis model assessment; INS, fasting insulin; LGB, laparoscopic adjustable gastric banding; REE, resting energy expenditure by metabolic cart; RYGB, Roux-en-Y gastric bypass; SAT, abdominal subcutaneous adiposity volume by computed tomography; SBP, systolic blood pressure; VAT, abdominal visceral adiposity volume by computed tomography; WC, waist circumference.

**Figure 1:**
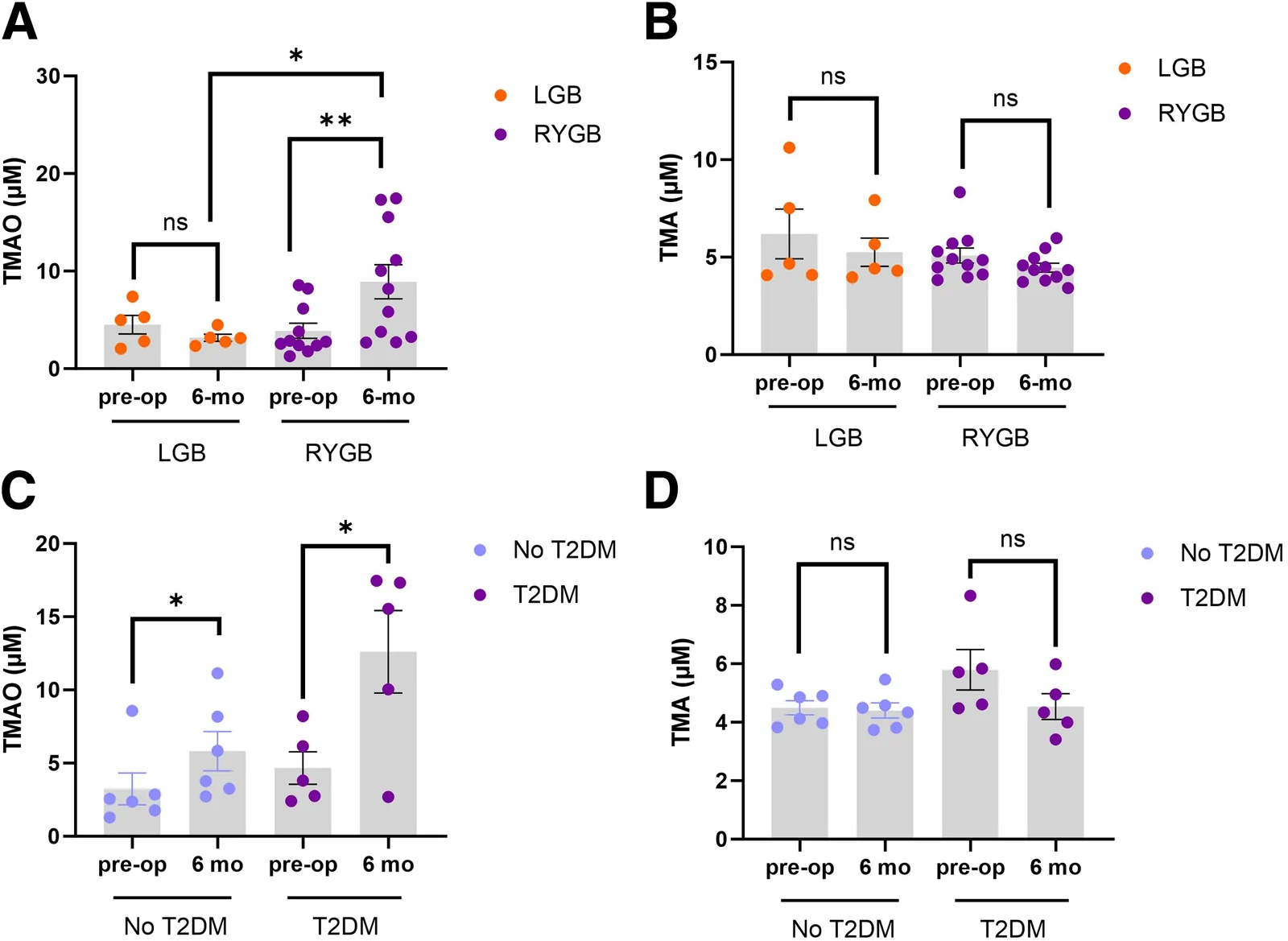
**Circulating methylamine concentrations in patients before and after bariatric surgery.** (*A*) Circulating TMAO and (*B*) TMA concentrations in patients undergoing RYGB or LGB. Normality was assessed using the Shapiro–Wilk test. Preoperative vs 6-month values were compared using a paired *t* test for the LGB group and a Wilcoxon matched-pairs signed-rank test for the RYGB group. At 6 months, the LGB and RYGB groups were compared using an unpaired *t* test. (*C*) Circulating TMAO concentrations in RYGB patients stratified by T2DM status. Preoperative vs 6-month values were compared using a Wilcoxon matched-pairs signed-rank test in the non-T2DM group and a paired *t* test in the T2DM group. (*D*) Circulating TMA concentrations in RYGB patients, compared between preoperative and 6-month time points using a paired *t* test. ∗*P* < .05, ∗∗*P* < .01. Data are presented in mean ± standard error of the mean.

Although TMAO has been reported to be positively associated with CRC risk,^17^^,^^18^ their causal relationship has not been elucidated. We, therefore, investigated the impact of TMAO on colonic tumor development using a Fabpl*Cre*;*Apc*^15lox/+^ mouse model.^23^ This genetically altered model predominantly develops tumors in the large intestine and closely mirrors familial adenomatous polyposis–associated CRC.^23^ Initially, we performed a baseline study to monitor the intestinal tumor onset and progression in mice fed with normal chow (NC) diet; this study was used to determine the duration of TMAO supplementation and the age of mice for tumor examination. Mice showed clinical symptoms (eg, blood in the stool) at an average age of 16.75 and 19.57 weeks for male and female mice, respectively. Female mice showed a longer survival compared with male mice (*P* = .0032), and no statistically significant differences in tumor load, gut length, colon length, and spleen weight were observed between male and female mice (Figure 2). Considering the sex-related phenotypic differences in tumor development, we selected 13 weeks of age for male mice and 15 weeks for female mice as the end timepoint (T2) to investigate the impact of TMAO on tumorigenesis in the early phase.

**Figure 2:**
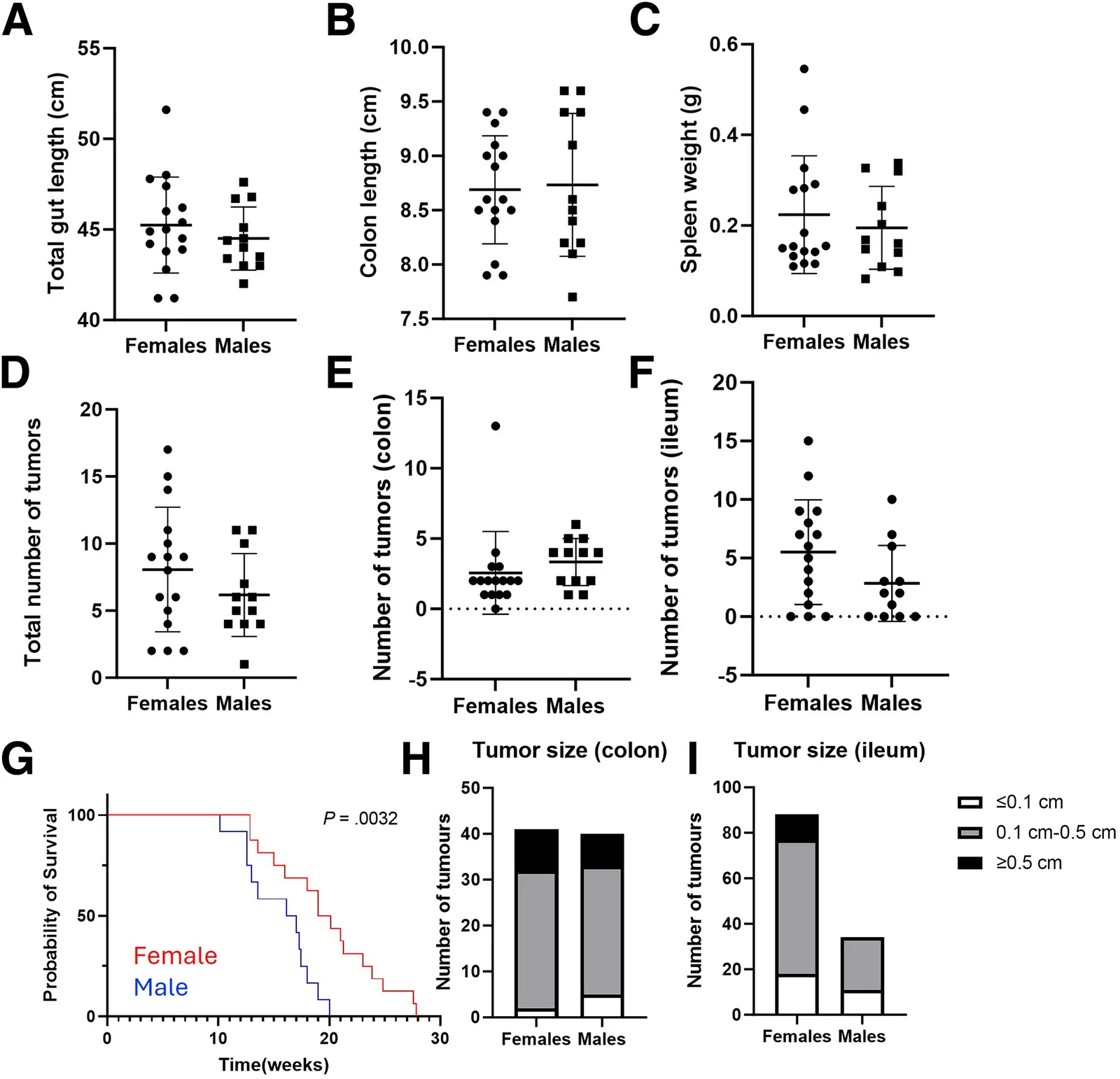
**Baseline phenotypic characteristics of Fabpl*Cre*;*Apc*^15lox/+^ mice.** (*A*) Total intestinal length, (*B*) colon length, (*C*) spleen weight, (*D*) total tumor number, (*E*) number of colonic tumors, and (*F*) number of ileal tumors in female and male mice. (*G*) Kaplan–Meier survival curves for female and male mice (Females n = 16, Males n = 12, *P* = .0032, log-rank test). Distribution of tumor sizes in the (*H*) colon and (*I*) ileum, categorized as ≤0.1 cm, 0.1–0.5 cm, or ≥0.5 cm. Data in (*A–F*) are presented as mean ± standard deviation.

Following 1-week acclimatization, mice at the age of 7 weeks old (T0) were fed either NC diet (control group) or NC supplemented with 0.12% (w/w) TMAO for 6 weeks in male mice and 8 weeks in female mice (Figure 3*A*). In addition, a midpoint (T1) was included to monitor anemia and TMAO levels in urine and plasma (Figure 3*A*). Although the male mice exhibited significantly higher body weight than the female mice, no significant differences observed between the control and TMAO groups of the same sex throughout the experiment (Figure 3*B*). The food intake did not differ between the male and female mice, nor between the control and TMAO groups (Figure 3*C*). Intestinal length and tumors were assessed at the end of the experiment; the lengths of the gastrointestinal tract and the colon did not differ between the control and TMAO groups (Figure 3*D* and *E*). However, a lower percentage of mice in the TMAO group developed colonic tumors compared with the control group, with a significant decrease observed in male mice (Figure 3*F*). Furthermore, tumor volume, taking both tumor count and size into the consideration, was significantly lower in the TMAO-treated male mice compared with the male controls, whereas this difference was not significant in female mice (Figure 3*G*). This observation was further supported by the histologic scores of hematoxylin and eosin (H&E) staining for the colonic tissues and tumors. Most of the male mice presented with no tumors in the TMAO group compared with the control group, unlike in female mice (Figure 3*H*). One of the clinical symptoms of these mice with intestinal tumors is anemia, which can be assessed using hematocrit.^26^^,^^27^ The hematocrit was significantly lower at T2 compared with T1, indicating the development of anemia (Figure 3*I*). These phenotypic observations together allow us to conclude that dietary supplementation with TMAO exerts a protective effect against colonic tumorigenesis in Fabpl*Cre*;*Apc*^15lox/+^ mice, particularly in male mice.

**Figure 3:**
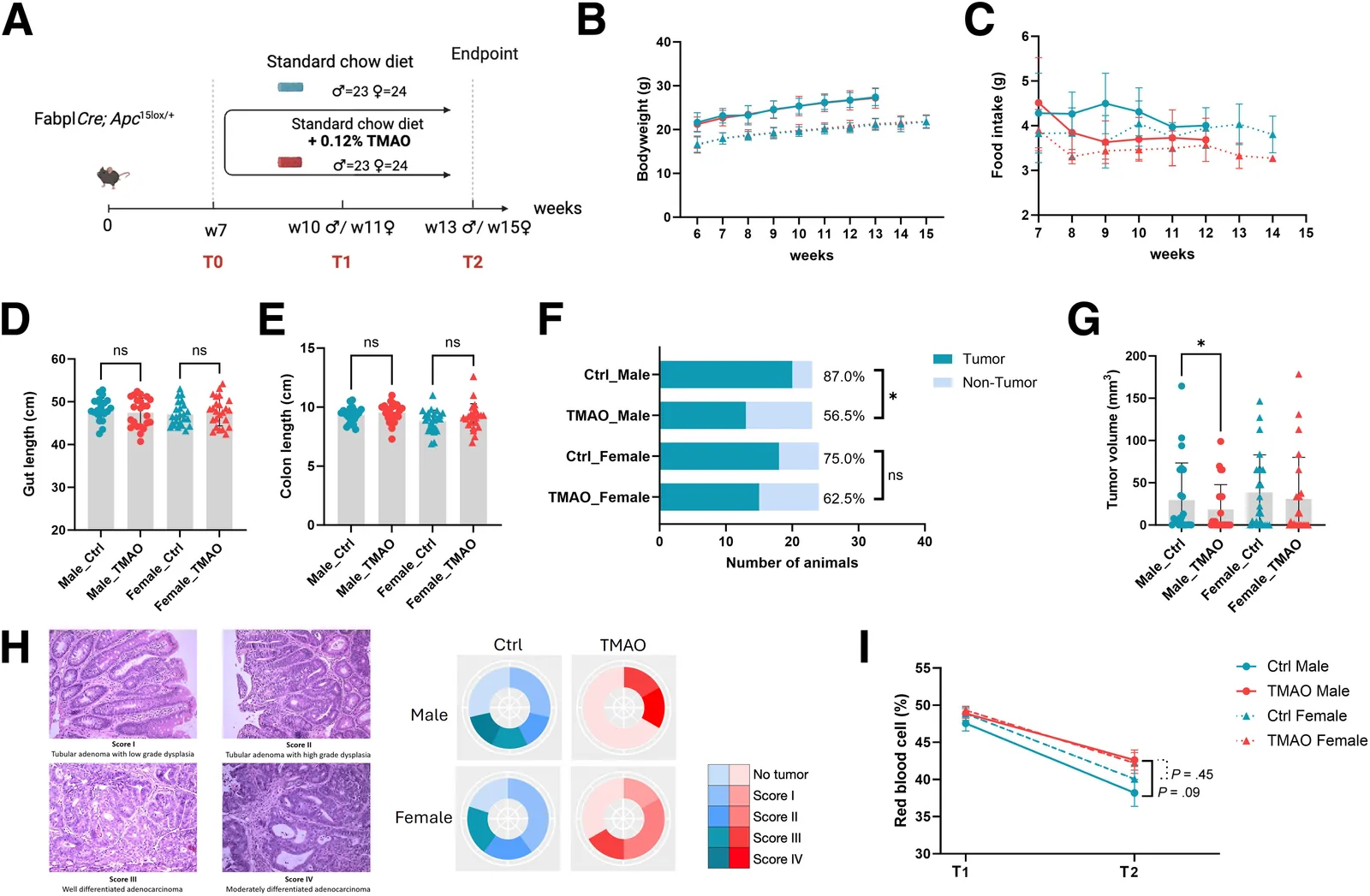
**Phenotypic characterization of Fabpl*Cre*;*Apc*^15lox/+^ mice with or without dietary TMAO supplementation.** (*A*) Experimental design of TMAO supplementation experiment using Fabpl*Cre*;*Apc*^15lox+^ mice. A total of 23 male and 24 female mice in the control group; and 23 male and 24 female mice in the TMAO group. T0, starting point at 7 weeks of age; T1, midpoint, 10 and 11 weeks of age; and T2, endpoint, 13 and 15 weeks of age for male and female mice, respectively. (*B*) Body weight changes between weeks 6 and 13 for males and weeks 6 to 15 for females. (*C*) Food intake. (*D*) Gut length and (*E*) colon length at the end of the experiment. (*F*) The proportion of mice that developed colonic tumors following either control or TMAO diet; statistical significance was assessed using the Fisher exact test. ∗*P* < .05. (*G*) total tumor volume per mouse in each group. Significance was determined by 1-way analysis of variance test. ∗*P* < .05. (*H*) Histologic grading of colonic tumors. *Left*, representative H&E-stained sections of colonic tumors illustrating the histopathologic scoring system used in this study. Score I: tubular adenoma with low-grade dysplasia; score II: tubular adenoma with high-grade dysplasia; score III: well-differentiated adenocarcinoma with preserved glandular architecture; score IV: moderately differentiated adenocarcinoma with increased architectural distortion and invasive features. Scale bars, 200 μm. *Right*, doughnut plots showing scores of tumor grading based on the histologic H&E staining for the colonic tissues and tumors from each group (5 male and 5 female mice in the control group; 6 male and 6 female mice in TMAO group). (*I*) Hematocrit values at T1 and T2 in controls and TMAO-supplemented mice. Data are shown as median and interquartile range as error bars. The slopes from T1 to T2 for all groups are statistically significant (control male mice *P* < .0001; TMAO male mice *P* = .0003; control female mice *P* = .0001; TMAO female mice *P* < .0001 by simple linear regression). At T2, male control vs TMAO, *P* =.09; female control vs TMAO, *P* = .45 by Dunn’s multiple comparisons test.

### The Bioavailability of Trimethylamine *N*-Oxide Following Dietary Supplementation Is Sex- and Supplementation Duration–Dependent

Given that the dietary supplementation of TMAO significantly reduced the colonic tumor load in male mice, we next explored if the bioavailability of TMAO following the dietary supplementation was sex dependent. The TMAO concentrations in the jejunum and ileum luminal contents were significantly higher in the TMAO group compared with the control group in both male and female mice (Figure 4*A*). However, this increase was not observed in the colon luminal content, suggesting that the absorption of dietary TMAO mainly occurs in the small intestine. No differences in the TMAO concentrations in the intestinal lumens were noted between male and female mice. Furthermore, no statistically significant differences in intestinal luminal TMA concentrations were observed (Figure 4*B*).

**Figure 4:**
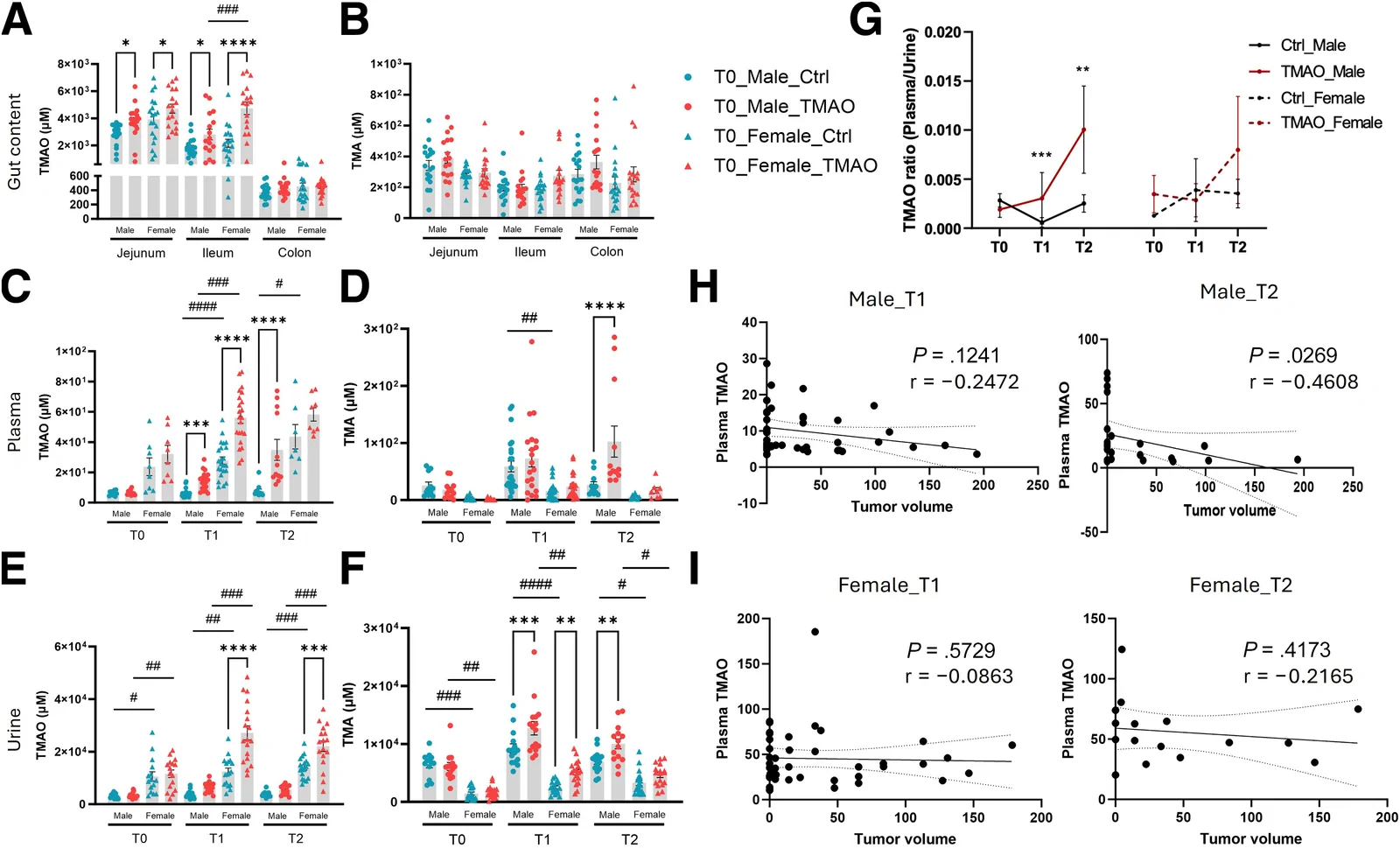
**Metabolic changes following dietary TMAO supplementation in male and female Fabpl*Cre*;*Apc*^15lox/+^ mice.** (*A*) TMAO and (*B*) TMA levels in jejunal, ileal, and colonic luminal contents. Urinary (*C*) TMAO and (*D*) TMA concentrations and plasma (*E*) TMAO and (*F*) TMA concentrations at T0, T1, and T2. (*G*) Plasma-to-urinary TMAO ratios at each time point. (*H* and *I*) Spearman correlation between circulating TMAO concentrations at T1 and T2 and tumor volume in (*H*) male and (*I*) female mice. Data are shown for control male mice (*blue circles*), TMAO-supplemented male mice (*red circles*), control female mice (*blue triangles*), and TMAO-supplemented female mice (*red triangles*). Statistical significance was assessed using the Kruskal–Wallis test (*B* and *C*) or 1-way analysis of variance (*A*, *D–F*). Comparisons in (*G*) were performed using the Student *t* test. ∗ and ^#^ indicate significant differences between treatment groups of the same sex, and between males and females subjected to the same treatment, respectively. ^∗ or #^*P* < .05, ^∗∗ or ##^*P* < .01, ^∗∗∗ or ###^*P* < .001, and ^∗∗∗∗ or ####^*P* < .0001. Data are presented as mean ± standard error of the mean.

Although circulating TMAO concentrations in female mice are intrinsically higher than the male mice as previously reported^28^ and as observed in our study (Figure 4*C*; T0 before the dietary supplementation), supplementation with TMAO for 3 or 4 weeks resulted in their significant increases in male and female mice, respectively (Figure 4*C*; T1). However, TMAO supplementation did not induce significant changes in TMA concentrations in the plasma at T1 (Figure 4*D*). Following supplementation with TMAO for 6 and 8 weeks (T2) in male and female mice, respectively, plasma TMAO remained elevated at T2 in male but not female mice (Figure 4*C*; T2). To further investigate this discordant trend, we measured TMAO excretion in the urine, where we observed that TMAO supplementation resulted in significantly higher concentrations of TMAO in female mice, but not in male mice (Figure 4*E*). The ratios of plasma to urinary TMAO levels were significantly higher in TMAO-supplemented male mice compared with the controls; however, this pattern was not observed in female mice (Figure 4*G*). Notably, male mice showed higher TMA excretion in the TMAO-treated group compared with the controls at both T1 and T2 (Figure 4*F*), which led us to conclude that there could be an enhanced retroconversion of TMAO to TMA through the gut bacterial activity^29^ following TMAO supplementation in male mice.

We next explored the correlation between the circulating concentrations of TMAO at T1 and T2 and the colonic tumor volume. Circulating TMAO levels measured at T1 and T2 were negatively correlated with the tumor volume in male mice, with a statistical significance (*P* = .0269; r = −0.4608) at T2 (Figure 4*H*). However, this correlation was not evident in female mice (Figure 4*I*). Additionally, TMAO supplementation did not induce significant shifts in the ^1^H nuclear magnetic resonance (NMR) spectroscopy–based global metabolic profiles of urine (Figure 5*A–D*), plasma (Figure 5*E–G*), and gut luminal contents (Figure 5I–K). However, it was noted that the metabolome of luminal contents from the jejunum, ileum, and colon were distinct (Figure 5*H*).

**Figure 5:**
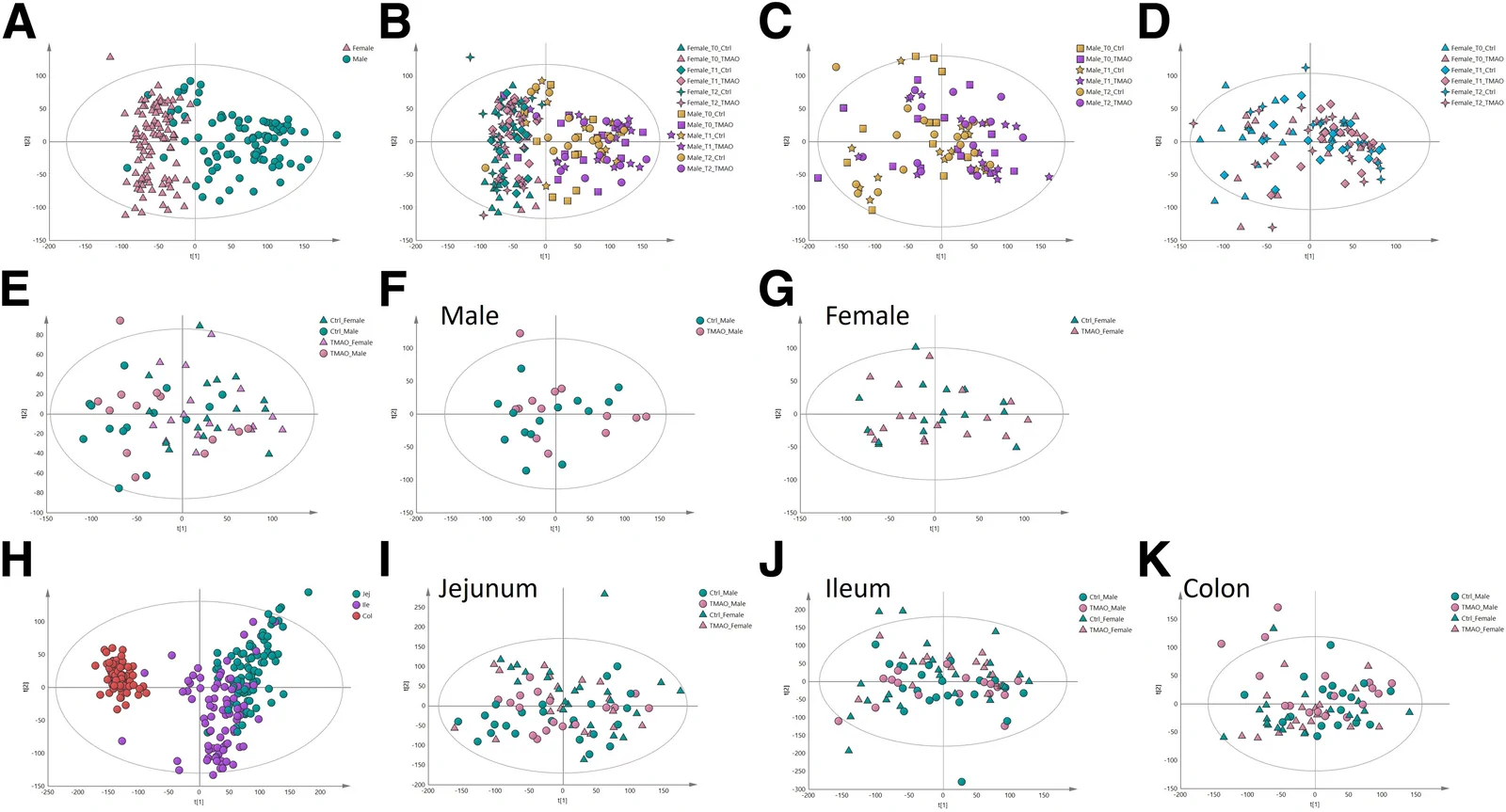
**Principal component analysis (PCA) of ^1^H NMR metabolic profiles from urine, plasma, and intestinal luminal contents of Fabpl*Cre*;*Apc*^15lox/+^ following dietary TMAO supplementation.** PCA scores plots are shown for (*A*) All urine samples colored by sex. Percentages of variation (R^2^X) explained by the first and the second principal components (PC1 and PC2) are 22.10% and 9.73%, respectively. (*B*) All urine samples colored by sex, timepoint, and treatment; R^2^X values of PC1 and PC2 are 22.10% and 9.73%, respectively. (*C* and *D*) Urine samples from male and female mice, respectively, colored by timepoints and treatments; R^2^X values of PC1 and PC2 are 23.90% and 11.70% in (*C*), and 13.10% and 7.82% in (*D*). (*E*) PCA scores plots from all plasma profiles; R^2^X values of PC1 and PC2 are 13.00% and 4.53%, respectively. (*F* and *G*) Plasma samples from male and female mice, respectively; R^2^X values of PC1 and PC2 are 14.10% and 7.30% in (*F*) and 11.80% and 5.82% in (*G*). PCA scores plots are shown for (*H*) all intestinal regions colored by anatomic locations and for (*I*) jejunal, (*J*) ileal, and (*K*) colonic contents colored by treatments. R^2^X values of PC1 and PC2 are 34.80% and 10.60% in (*H*), 20.40% and 17.50% in (*I*), 20.30% and 19.60% in (*J*), and 15.10% and 8.45% in (*K*), respectively. Data were mean-centered and unit variance-scaled prior to PCA.

These findings let us to conclude that absorption of dietary TMAO mainly occurred in the small intestine in both male and female mice. Although TMAO supplementation increased the circulating TMAO levels in both male and female mice at the midpoint T1, the retention of TMAO at the endpoint T2 was higher in male mice compared with female mice. This higher retention of TMAO was found to be significantly inversely associated with the colonic tumor load in male mice.

### Dietary Supplementation of Trimethylamine *N*-Oxide Altered the Colonic Immune Landscape and Significantly Lowered the Percentages of Tumor Necrosis Factor-α–Expressing Intraepithelial Lymphocytes in Male Mice

To further investigate the potential mechanism through which TMAO supplementation induced antitumorigenic responses in Fabpl*Cre*;*Apc*^15lox/+^ mice, we assessed the landscape of immune cell subsets within the colonic intraepithelial lymphocytes (IELs) and lamina propria lymphocytes (LPLs) using flow cytometry. As we focused on the effect of TMAO on CRC risk, we analyzed IELs and LPLs instead of tumors, which represent tumor microenvironment–related immune responses. The initial observation revealed a higher percentage of the total CD45^+^ cells in IELs of male mice receiving TMAO supplementation compared with the controls, although the statistical significance was not reached (Figure 6*A*). We concluded that there was a potential increase in immune cell infiltration, indicating a heightened immune response, in response to elevated concentrations of TMAO. In both the control and TMAO groups, CD8+ T cells and γδT cells dominated in the IELs, whereas myeloid cells, CD4+ T cells, and natural killer (NK) cells were predominant in the LPLs (Figure 6*B*). When comparing the TMAO group with the controls, CD8+ T cell frequencies were higher in the LPLs of both male and female mice. In contrast, innate lymphoid cell 1 (ILC1) frequencies were higher in both IELs and LPLs from female mice fed with TMAO supplementation compared with the control group, but this trend was not observed in male mice (Figure 6*C*). Furthermore, TMAO supplementation in male mice increased the frequencies of NK cells in both the IELs and LPLs, whereas in female mice, it led to a decrease (Figure 6*C*). In addition, the ratio of CD8+ to FoxP3+ was calculated because it has been reported as an indicator of the balance between cytotoxic T cells and regulatory T cells. A high ratio of CD8+ to FoxP3+ in the tumor microenvironment has been associated with improved outcomes in different types of cancer, such as breast cancer and CRC.^30^^,^^31^ In our study, we observed a slight increased CD8^+^:Foxp3^+^ ratio in the colonic mucosa of TMAO-fed male mice (Figure 6*D*), potentially suggesting a shift toward a more cytotoxic and less immunosuppressive immune profile. This balance suggests that TMAO may enhance local antitumor immunity by promoting effector T-cell activity while limiting regulatory T-cell–mediated suppression.

**Figure 6:**
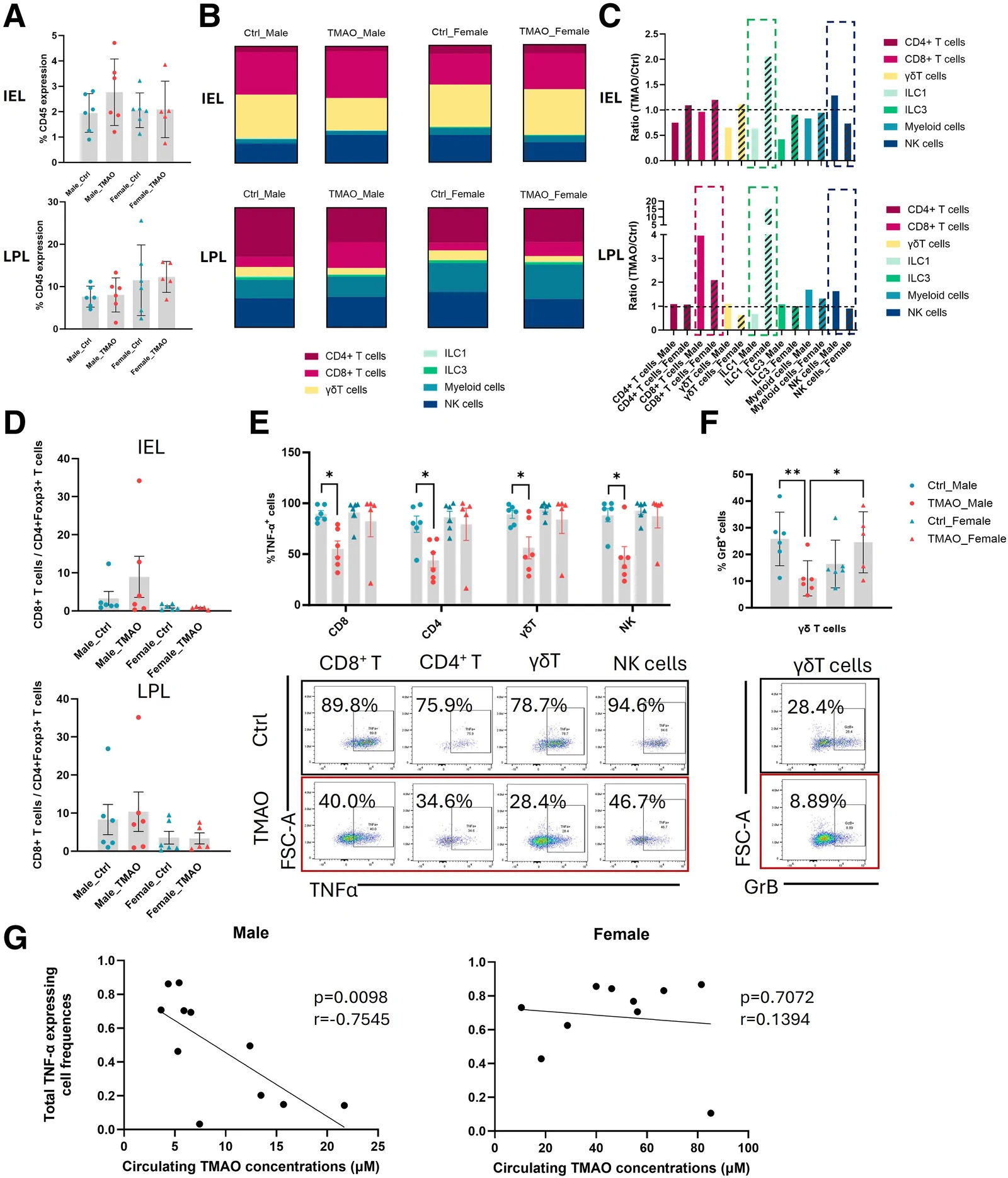
**Immune profiles of colonic IELs and LPLs from Fabpl*Cre*;*Apc*^15lox+^ mice fed with NC (controls, Ctrl) or NC supplemented with TMAO.** (*A*) Percentages of CD45^+^ cells in the colonic IELs and LPLs. Statistical significance was assessed using the Kruskal–Wallis test. (*B*) Composition of immune cell subsets in IELs and LPLs from control and TMAO-supplemented groups. Cells were gated on nonautofluorescence lymphocytes with subsequent gating as follows: CD4+ T cells: CD3+CD4+; CD8+Tcells: CD3+CD8+; γδT cells: CD3+TCRγδ+; Myeloid cells: CD3−CD19−NK1.1−CD11b+ and NK cells: CD3-NK1.1+. (*C*) Fold changes (ratios) in immune cell subset frequencies in TMAO-supplemented mice relative to controls. (*D*) Ratios of CD8+ T cells to Foxp3+ cells in colonic IEL and LPL compartments. (*E*) Percentages of TNF-α–expressing cells within each subset (CD8+, CD4+, γδT, and NK cells) in the IELs and representative gating strategies in flow cytometry analysis. (*F*) Percentages of GrB+-expressing cells within total γδT cells in IELs and representative gating strategies in flow cytometry analysis. (*G*) Spearman’s correlation analysis between circulating TMAO levels at T1 and TNF-α–expressing cell frequences in the total nonautofluorescent CD45+ cells. Significance was determined by Kruskal–Wallis test. ∗*P* < .05, ∗*P* < .01. Data are presented as mean ± standard error of the mean, n = 6 per group per sex.

Following the compositional analysis of immune cell subsets, we investigated the TMAO-induced changes at the functional level, and these included CD107a, granzyme B (GrB), interferon-γ, interleukin (IL)17, NK group 2 member D, programmed cell death protein 1, and tumor necrosis factor-α (TNF-α). We observed that TMAO supplementation in male mice significantly lowered percentages of TNF-α–producing CD4+ T, CD8+ T, γδT, and NK cells in the IELs (Figure 6*E*). In addition, other TNF-α-expressing innate immune cells within the IELs, including CD11b+Ly6C-Ly6G+ polymorphonuclear–myeloid-derived suppressor cells (PMN-MDSCs) and ILC1, showed a lower trend in males with TMAO supplementation compared to controls, but this was not statistically significant (Figure 7*A* and *B*). Furthermore, TMAO supplementation reduced the percentage of GrB-producing γδT cells in the IELs of male mice, but not in female mice (Figure 6*F*). Total TNF-α–expressing cell frequencies were significantly and negatively correlated with circulating TMAO concentrations in male mice (*P* = .0098; r = −0.7545) (Figure 6*G*) but not in female mice (Figure 6*F*). These changes observed in the colonic IELs were not noted in the colonic LPLs (Figure 7*C−E*). The immune profiles of LPLs and IELs from the duodenum–jejunum and the ileum showed no evident differences between the control and TMAO groups (Figure 8). However, it was noted in the duodenum–jejunum LPLs that the ILC1 ratio between the TMAO and control mice was higher in female mice (ratio, 2.38) compared with male mice (ratio, 0.64) (Figure 8*F*), but this trend was reversed in the ileum (female: ratio, 0.09; male: ratio, 6.52) (Figure 8*L*).

**Figure 7:**
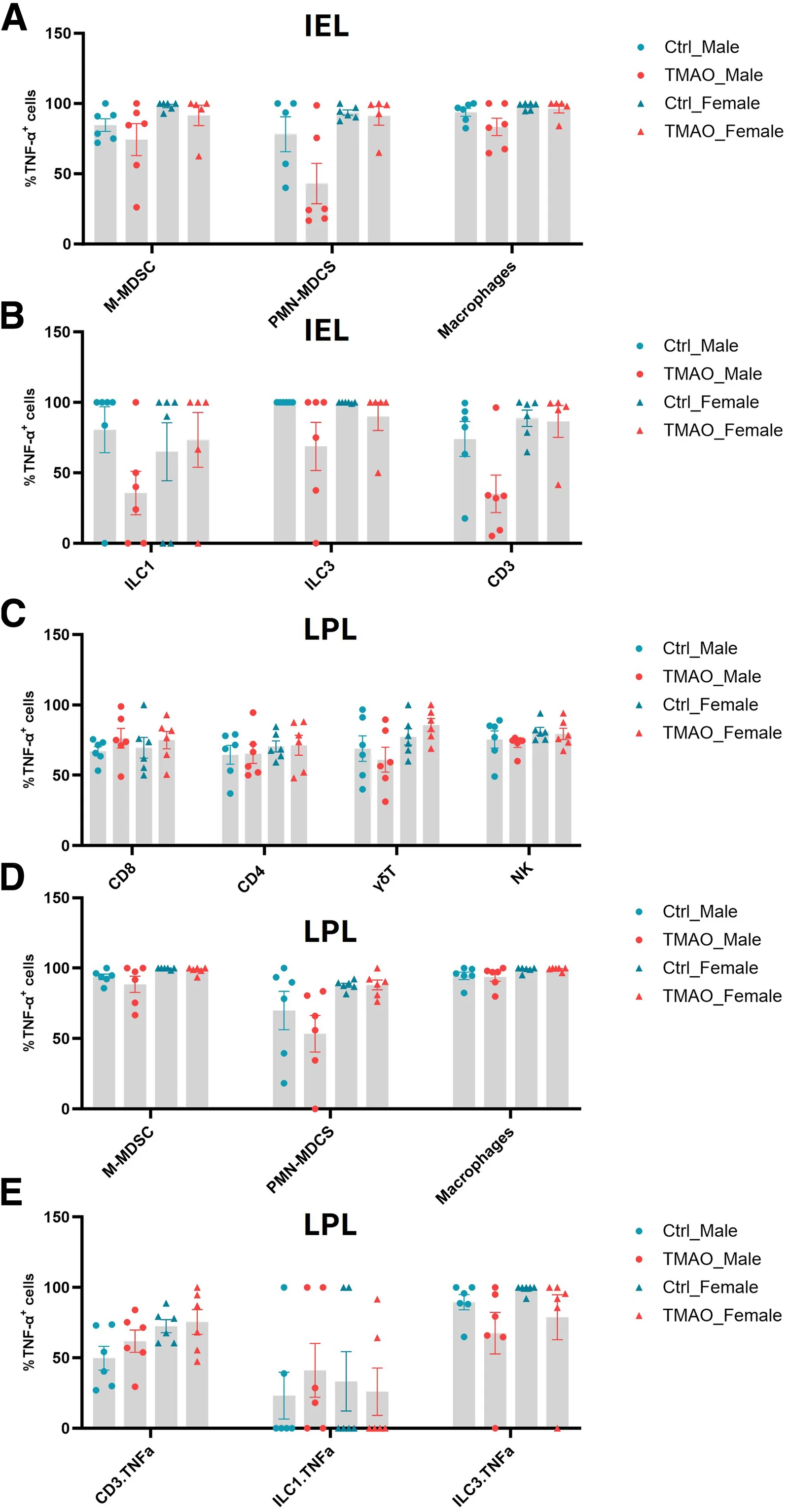
**TNF-α expression in colonic IEL and LPL populations following dietary TMAO supplementation.** (*A* and *B*) Frequencies of TNF-α+ cells within immune cell subsets in colonic IELs from Fabpl*Cre*;*Apc*^15lox+^ mice fed with NC (controls, Ctrl) or NC supplemented with TMAO. (*C–E*) Frequencies of TNF-α+ cells within immune cell subsets in colonic LPLs from controls and TMAO-supplemented mice. Statistical significance was assessed using the Kruskal–Wallis test. Data are presented in mean ± standard error of the mean, n = 6 per group per sex.

**Figure 8:**
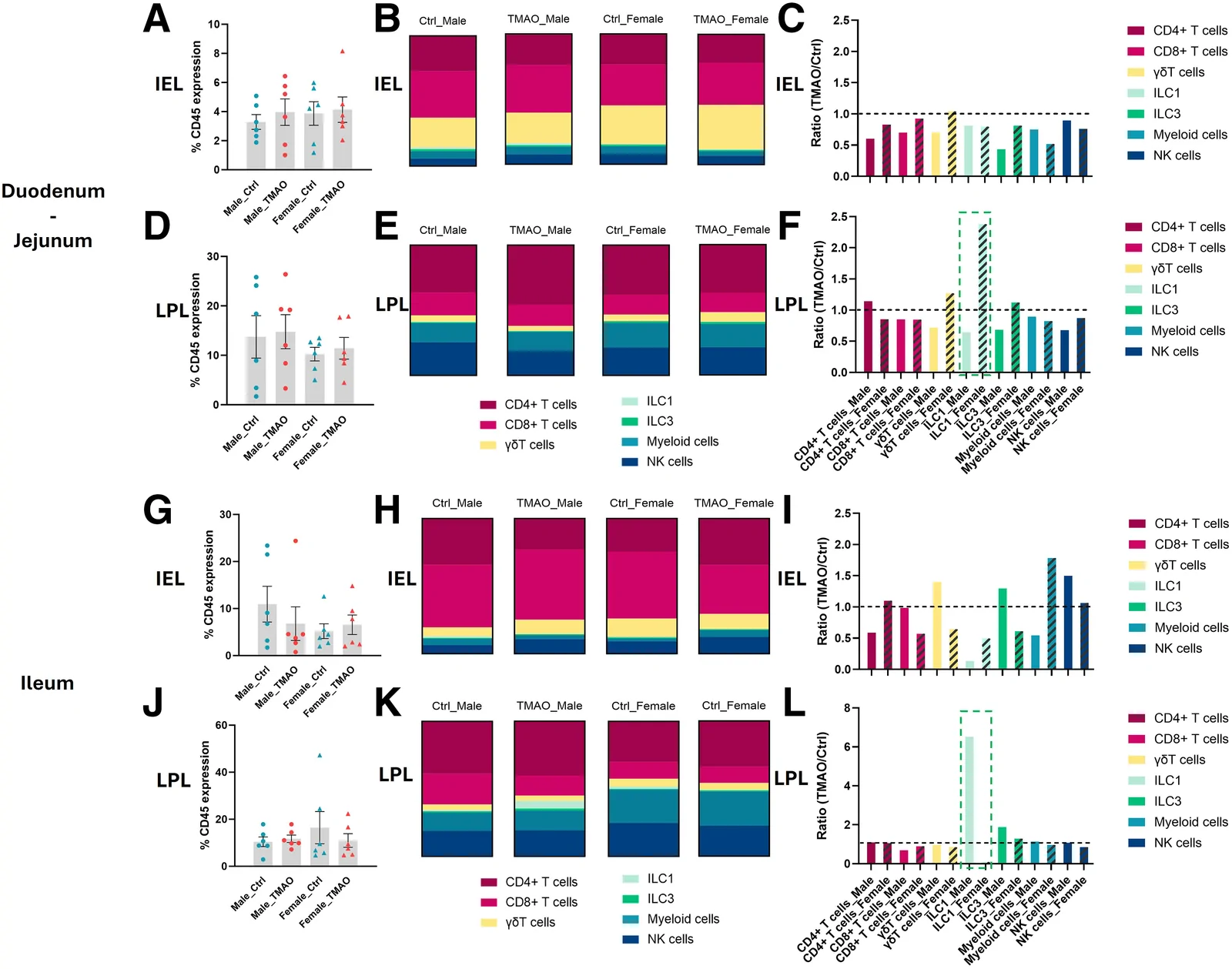
**Immune profiles of duodenum-jejunum and ileal IELs and LPLs following dietary TMAO supplementation.** (*A–F*) Immune profiles of duodenum-jejunum IELs and LPLs from control and TMAO-supplemented Fabpl*Cre*;*Apc*^15lox+^ mice. (*A* and *D*) Frequencies of CD45+ cells in IEL and LPL compartments. (*B* and *E*) Composition of immune cell subsets in IELs and LPLs. Immune populations were identified following exclusion of auto-fluorescent cells and defined as follows: CD4+ T cells, CD3+CD4+; CD8+ T cells, CD3+CD8+; γδ T cells, CD3+TCRγδ+; myeloid cells, CD3−CD19−NK1.1−CD11b+; and NK cells, CD3−NK1.1+. (*C* and *F*) Fold changes in immune cell subset frequencies in TMAO-supplemented mice relative to controls. (*G–L*) Immune profiles of ileal IELs and LPLs. (*G* and *J*) Frequencies of CD45+ cells; (*H* and *K*) composition of immune cell subsets; and (*I* and *L*) fold changes in immune cell subset frequencies in TMAO-supplemented mice relative to controls. Statistical significance was assessed using the Kruskal–Wallis test. Data are presented as mean ± standard error of the mean; n = 6 mice per treatment group per sex.

Collectively, our data demonstrated that dietary supplementation with TMAO reduced the percentages of TNF-α–expressing CD4+ T cells, CD8+ T cells, γδT, and NK cells in the colonic IELs of male mice, favoring a reduced inflammatory environment in the colon to reduce tumorigenesis.

### Trimethylamine *N*-Oxide Reduces Tumor Necrosis Factor-α Levels in Lipopolysaccharide-Stimulated Murine Macrophages

Following our observation that dietary supplementation of TMAO reduced TNF-α–expressing immune cell subsets in the colonic IELs in male mice, we investigated the direct impact of TMAO on TNF-α secretion from lipopolysaccharide (LPS)-stimulated murine macrophages (RAW264.7 cell line originally established from a male mouse). We first confirmed that TMAO and TMA at the concentration ranges of 5 μM to 500 μM and 5 μM to 150 μM, respectively, did not induce cytotoxic effects (Figure 9*A–D*). We next chose physiological circulating concentrations of TMAO (50 μM) and TMA (150 μM) observed in the male mice at T2 (Figure 4*C*) to prime cells for 24 hours and exposed the cells to LPS (10 ng/mL) for 16 hours. Both TMAO and TMA significantly suppressed TNF-α secretion (Figure 9*E*), which is consistent with our observations in vivo. Furthermore, TMAO exposure at a concentration of 50 μM for 24 hours to human colon cancer HCT116 cells did not induce cell proliferation (Ki67+) and genotoxicity (γH2AX) (Figure 10), and we suggest that TMAO did not increase colonic cancer risk factors.

**Figure 9:**
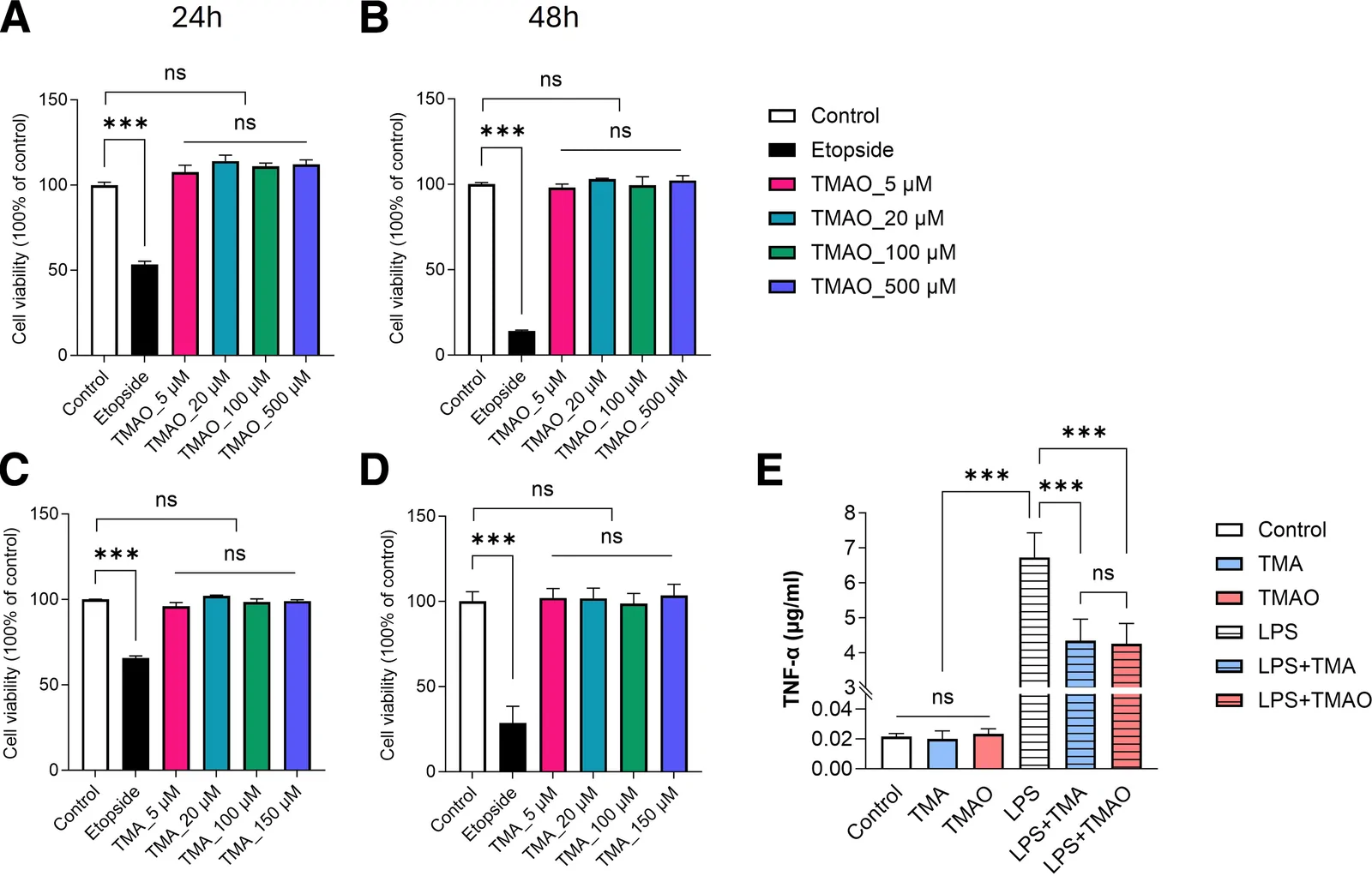
**Effects of TMAO and TMA on RAW264.7 macrophage viability and TNF-α production.** (*A–D*) Viability of RAW264.7 macrophages following treatment with TMAO of 5, 20, 100, and 500 μM for 24 hours (*A*) and 48 hours (*B*), or TMA at concentrations of 5, 20, 100, and 150 μM for 24 hours (*C*) and 48 hours (*D*), assessed using the AlamarBlue assay. Cell viability is expressed relative to untreated controls. (*E*) TNF-α concentrations in culture supernatants from RAW264.7 cells pretreated with TMAO (50 μM) or TMA (150 μM) for 24 hours, followed by LPS stimulation (10 ng/mL) for 16 hours. Statistical significance was assessed using 1-way analysis of variance. ∗∗∗*P* < .001. ns, no significance. Data are presented as mean ± standard error of the mean; 3 independent biological replicates per treatment group.

**Figure 10:**
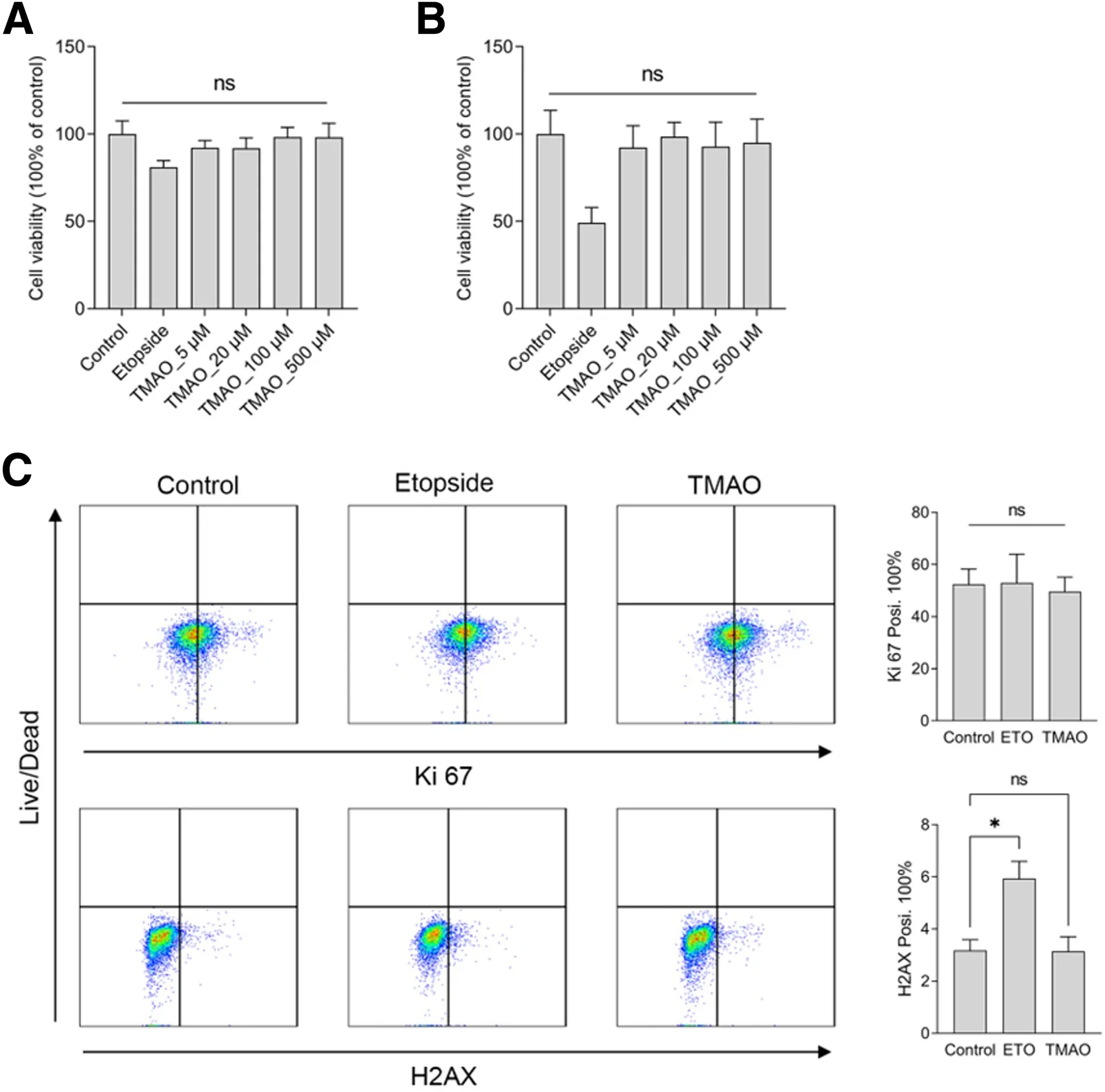
**Cytotoxic and genotoxicity effects of TMAO on human colonic HCT116 cells.** Cells were treated with TMAO at concentrations of 5, 20, 100, and 500 μM for (*A*) 24 hours and (*B*) 48 hours. Cell viability following TMAO treatment was determined by AlamarBlue assay. Cell viability is expressed relative to untreated controls. (*C*) Expression levels of Ki-67 and γH2AX staining in HCT116 cells following 50 μM TMAO treatment for 24 hours, assessed by flow cytometry. Data is expressed relative to untreated controls. Etoposide (ETO) served as positive control. ∗*P* < .05; ns, not significant. Data are presented as mean ± standard error of the mean from 3 independent biological replicates.

## Discussion

Consistent with previously published findings,^7^ we observed that TMAO circulating concentrations significantly increased 6 months post-RYGB surgery, but not after LGB. Although it was reported that circulating TMAO increased more than 2-fold in patients with T2DM after RYGB surgery, but not in patients without T2DM,^5^ we found that both groups of patients exhibited significant increases, with a higher degree observed in patients with T2DM. In contrast, TMA levels remained similar before and after the surgery. In a long-term follow-up study, circulating levels of TMAO have remained significantly higher 8 years post-RYGB.^7^

In the current study, we directly demonstrated the protective effects of dietary TMAO against colonic tumor development in a sex-dependent manner using a genetically altered murine model, Fabpl*Cre;Apc*^*15lox/+*^ mice, which develops colonic tumors and better mimics human sporadic CRC. We further reported that plasma TMAO concentrations, resulting from dietary TMAO supplementation, were negatively correlated with the tumor load in male, but not female, mice. This protective role of TMAO has not previously been reported in CRC, but lower serum levels of TMAO were observed in patients with pancreatic cancer.^32^ Furthermore, TMAO was recently shown to reduce tumor growth in mice with orthotopic pancreatic ductal adenocarcinoma.^22^ Mirji and colleagues reported that delivering TMAO intraperitoneally or via a dietary supplement to orthotopic pancreatic ductal adenocarcinoma–bearing mice reduced their tumor growth, which was associated with an immunostimulatory tumor-associated macrophage phenotype, and activated effector T cell response in the tumor microenvironment.^22^ Another study demonstrated that patients with triple-negative breast cancer with higher plasma TMAO achieved better responses to immunotherapy, and TMAO induced tumor cell pyroptosis by activating protein kinase R–like endoplasmic reticulum kinase and enhanced CD8+ T-cell–mediated antitumor immunity.^21^ These findings are in line with our observation that higher circulating levels of TMAO in male mice exert a protective role against colonic tumor development in vivo and provide a rationale to explore the immune and inflammatory effects of TMAO in CRC. However, a recent study in *Apc*^min/+^ female mice reported that orally administered TMAO promoted intestinal tumorigenesis by suppressing the farnesoid X receptor–fibroblast growth factor 15 axis. These findings indicate that effects of TMAO on CRC are context dependent, varying with experimental model, sex, and exposure conditions.^33^

We revealed a new role of dietary TMAO in modulating colonic immune subsets; dietary TMAO significantly reduced the frequencies of TNF-α–expressing CD4+ and CD8+ T cells, and γδT and NK cells in the colonic IELs of male mice. TNF-α is a known proinflammatory cytokine that is associated with tumor promotion by bridging inflammation and carcinogenesis.^34^ Specifically, it stimulates the production of other proinflammatory cytokines, such as IL6 and IL8, which contribute to the inflammatory microenvironment associated with CRC.^35^ Furthermore, we found that the circulating TMAO concentrations were negatively associated with the total TNF-α–expressing immune populations, and exposure of TMAO to murine macrophage RAW264.7 cells significantly reduced LPS-stimulated TNF-α production. From this evidence, we propose that higher circulating TMAO concentrations could suppress tumorigenesis through reducing TNF-α–expressing immune subsets. Blocking TNF-α signaling or using TNF-α knockout mice markedly suppresses intestinal tumor development^36^^,^^37^; hence, TNF-α signaling may be a promising target for reducing obesity-associated CRC risk.^36^ Following RYGB, increased TMAO levels may exert a beneficial immunomodulatory effect; however, this possibility requires further investigation in cohorts of patients who have undergone RYGB.

Our rodent experiment with dietary TMAO supplementation also showed compelling evidence that the dietary TMAO was mainly absorbed in the jejunum and ileum, circulating TMAO levels were affected by its urinary excretion, and the duration of supplementation dynamically affected the circulating TMAO levels in female mice. We revealed that urinary excretion of TMAO was not affected by dietary supplementation in male mice but was significantly increased in female mice; as a result, a higher retention of blood TMAO in male mice compared with female mice was observed. It was estimated that 50% of ingested TMAO remains unchanged and excreted from urine and the other 50% undergoes retro-conversion to TMA and is reoxidized to TMAO before urinary excretion.^38^ We observed an increase in circulating TMA in male mice at the final time point, but not in female mice, which could be explained by previously reported higher flavin-containing monooxygenase 3 activities oxidizing TMA to TMAO in female mice.^28^^,^^39^

In summary, our novel findings suggest that circulating TMAO plays a protective effect against colonic tumor growth through downregulating TNF-α–expressing cell frequencies. This study contributes to the growing interest in CRC risk following bariatric surgery and the role of TMAO in modulating CRC risk, and it provides a new perspective on the relationship between dietary TMAO intake and circulating TMAO levels, highlighting the importance of circulating TMAO retention in CRC prevention.

### Limitations of the Study

Although our study provided evidence supporting the causal relationship between TMAO and CRC risk, several limitations can be addressed in future studies. We quantified the circulating TMAO and TMA levels in patients at 6 months post-RYGB or -LGB. Long-term follow-up studies with longitudinal collection of paired urine and blood samples will be insightful for understanding TMAO circulation and retention post-bariatric surgery. Nevertheless, our findings highlight the importance of TMAO retention in blood in CRC development. The effect of TMAO on colonic tumorigenesis was assessed using Fabpl*Cre;Apc*^*15lox/+*^ mice in the current study. We chose this model as it has been shown to better mimic the human sporadic CRC compared with *Apc*^min/+^ mice.^23^ As we highlighted a role of TMAO on downregulating TNF-α–expressing cell frequencies in colonic IELs, it would be interesting to further investigate the role of TMAO in TNF-α knockout mice. TNF-α knockout has been reported to attenuate the high-fat diet–associated increase in intestinal tumorigenesis.^36^ Female mice generally exhibit higher circulating and urinary TMAO and lower TMA levels compared with male mice. In our study, we monitored the temporal changes in TMAO and TMA levels, which prompted us to conclude that male mice demonstrated a higher retention of circulating TMAO. Here, we cannot preclude the potential effects of TMA on intestinal tumorigenesis and the downregulation of TNF-α–expressing cell frequencies in colonic IELs. Further investigation is warranted to distinguish the separate effects of TMAO and TMA.

## Materials and Methods

### Ethics Approval and Consent to Participate

Serum samples were collected from 16 patients recruited from Emory University, USA (ethics reference: Institutional Review Board #333-2002). The animal study was carried out under the Animals (Scientific Procedures) Act 1986 and received ethical approval (Project License: 9718F9C8) from the UK Home Office.

### Patient Cohort

A total of 16 patients (undergoing LGB [n = 5] or RYGB [n = 11]) were recruited from Emory University (Institutional Review Board #333-2002). Serum samples were collected preoperatively (pre-op) and 6 months after surgery (post-op), as previously detailed.^24^

### Fabpl*Cre;Apc*^*15lox/+*^ Mouse Experimental Design

The animal study was conducted in accordance with the UK Home Office Animals Scientific Procedures Act 1986 and the EU Directive 2010, with a UK Home Office project license of PPL P9718F9C8. Fabpl*Cre;Apc*^*15lox/+*^ mice^23^ were generated by crossing *Apc*^*15lox/15lox*^ (JAX stock #029275) with homozygous Fabpl *Cre* mice in the animal facility at Imperial College London. Mice were maintained under specific pathogen-free conditions on a 12-hour light-dark cycle at a temperature of 21 ± 1 °C. Their health status was monitored daily to ensure their welfare.

A preliminary baseline study was conducted to determine the onset of intestinal tumorigenesis and establish comparable experimental end points between sexes in Fabpl*Cre;Apc*^*15lox/+*^ mice. We observed that male mice began developing tumors 2 weeks earlier than female mice (at 13 weeks for male and 15 weeks for female mice). Therefore, the mice were sacrificed at these ages in the following TMAO experiment.

A total of 46 male and 48 female 6-week-old Fabpl*Cre;Apc*^*15lox/+*^ mice were included in the TMAO supplementation experiment. Six-week-old mice were acclimatized in the facility for 1 week with free access to water and NC diet (Product code 1535-00, Ssniff). Following acclimatization, mice were randomized into 2 groups: control group (controls) fed on NC, and the TMAO group fed on the same NC, but containing 0.12% (w/w) TMAO (Product code S9468-P020, Ssniff). To minimize potential bias, mice from the same breeding pairs with comparable body weights were allocated to different treatment groups. This supplementation dose of 6 mg TMAO per day (0.12% × 5 g of daily food intake (equivalent to 15 kcal/day) has previously been used to study the impact of TMAO on cardiovascular diseases.^40^^,^^41^ The dose corresponds to an individual’s daily intake of 800 mg of TMAO per 2000 kcal (a factor of 133.3 in calorie intake), and this intake level is equivalent to consuming approximately 200 g of red bream fish.^42^^,^^43^ At the age of 7 weeks post-acclimatization, male and female mice were subjected to 6- and 8-week supplementation of TMAO in their diet.

Blood samples were collected by tail venipuncture at baseline (T0; after acclimatization, before the group assignment), midpoint (T1; at the middle period of dietary intervention, age of 10 weeks for males and 11 weeks for females) and endpoint (T2, at the end of dietary intervention, age of 13 weeks for males and 15 weeks for females). Blood was collected into heparin-coated capillary tubes and centrifuged at 11,200 *g* for 3 minutes to obtain plasma. Hematocrit was calculated as the proportion of packed red blood cells within the total blood column to assess anemia.^44^ Urine and fecal samples were collected for metabolomics studies at T0, T1 and T2. Biofluid samples were stored in a −80 °C freezer pending analysis.

Mice were sacrificed at the age of 13 and 15 weeks for male and female mice, respectively, to collect the gut luminal contents. Researchers were blind to the mouse groups. After the removal of the cecum, the small intestine was divided into 3 equal parts: duodenum, jejunum, and ileum. After removing the remaining fat, the content from each part of the intestine was collected. The gut was opened carefully to avoid cutting through tumors, and tumors were counted, and the size was measured. The tumor volume was calculated using the following formula: (4/3) × π × (diameter of each tumor/2).^3^

### Histologic Analysis of Intestinal Tumors

Histologic analysis of tumor samples involved fixation in 4% v/v formaldehyde solution, followed by staining with H&E. This staining method allows for visualization of tissue structure, cell distribution, and morphologic changes. A blind evaluation of the stained slides was carried out by a pathologist. Tumor scoring was based on the following criteria: score I: tubular adenoma with low-grade dysplasia; score II: tubular adenoma with high-grade dysplasia; score III: tubular adenoma with high-grade dysplasia or well-differentiated adenocarcinoma; and score IV: tubular adenoma with severe dysplasia or moderately differentiated adenocarcinoma. Representative images of H&E-stained sections of colon tumors with scores I to IV are shown in Figure 3*H*.

### ^1^H Nuclear Magnetic Resonance Spectroscopy-Based Metabolic Profiling of Urine, Plasma, and Gut Contents

^1^H NMR spectroscopy was applied to generate global metabolic profiles of urine, plasma, and gut content samples.^45^ Urine and plasma samples were thawed at room temperature (21 ± 1 °C) and spun at 20,000 *g*, 4 °C for 10 minutes. For urine analysis, a total of 25 μL of the supernatant from each sample was mixed with 34 μL of deuterium oxide (purity 99.99%; Merck) and 6 μL of potassium phosphate buffer.^45^ The urine mixture was briefly spun and 60 μL was transferred to an NMR tube with an outer diameter of 1.7 mm using an automatic syringe pipette (SGE eVol Handheld Automated Analytical Syringe). For plasma analysis, a total of 25 μL of the supernatant from each sample was mixed with 15 μL deuterium oxide (purity 99.99%, Merck) and 40 μL of plasma potassium phosphate buffer,^45^ and 60 μL of the mixture was transferred to a 1.7-mm NMR tube.

Approximately 150 mg of gut content from the jejunum, ileum, and colon were weighed, and 2 portions of HPLC water (eg, adding 200 μL to 100 mg of content) were added to the sample. The pellets were vortexed for 5 minutes and centrifuged at 20,000 *g* for 10 minutes at 4 °C. A volume of 180 μL of the supernatant was transferred to a new 1.5-mL microcentrifuge tube containing 20 μL of the potassium phosphate buffer.^45^ The mixture was vortexed for 5 seconds and briefly spun. A total of 180 μL of the supernatant was transferred to a 3-mm NMR tube for ^1^H NMR spectroscopic analysis.

^1^H NMR spectra of medium, urine and gut content samples were obtained using a 600 MHz ^1^H NMR spectrometer Bruker AVANCE III (Bruker), operating ^1^H frequency at 600.13 MHz at a temperature of 300 K for urine and gut content, and 310 K for plasma. A standard NMR pulse sequence was applied (1-dimensional Nuclear Overhauler Enhancement Spectroscopy: preset (noesypr1d): relaxation delay-90°-t_1_-90°-t_m_ (mixing time)-90°-acquisition) to acquire 1-dimensional spectral data. The spectral width is 20 ppm, and 4 dummy scans were used. A total of 64 scans were collected into 20k data points for urine, gut content, and plasma. Phase correction, baseline correction, and calibration to the singlet from 3-(trimethylsilyl)propionic-2,2,3,3-d_4_ acid sodium salt (TSP) at 0.00 ppm were manually performed in TopSpin 3.1 (Bruker). ^1^H NMR spectra were imported to MATLAB (R2014a) software. The peak regions of TSP (δ^1^H, −0.2 to 0.2) and water (δ^1^H, 4.55–4.80) were removed. The probabilistic quotient method was applied to normalize the remaining spectra.^46^ Principal component analysis was constructed on the mean-centered and unit variance–scaled data in SIMCA (13.0).

TMAO and TMA in intestinal contents and urine were identified using Chenomx NMR suite 8.3 software (Chenomx Inc). The peaks of TMA (singlet at 2.88 ppm; range, 2.8760–2.8910 ppm), TMAO (singlet around 3.26 ppm; range, 3.2550–3.2800 ppm), and TSP (singlet at 0 ppm; range, −0.2000 to 0.2000 ppm) were integrated from gut content and urinary spectra, and the concentrations of TMA and TMAO were calculated based on the concentration of TSP using the following equation:

$$TMAO\;concentration=\frac{\Delta integrals\;of\;TMAO\;peak}{\Delta integrals\;of\;TSP\;peak}\times TSP\;concentration$$

### Quantification of Trimethylamine *N*-Oxide and Trimethylamine Using a Targeted Ultra-Performance Liquid Chromatography–Tandem Mass Spectrometry

TMAO and TMA concentrations in patient serum and mouse plasma were quantified in patient serum samples, and mouse plasma samples were quantified using a previously established targeted UPLC–MS/MS method.^25^ Briefly, 10 μL of the sample was mixed with 10 μL of the internal standard solution containing TMA-d_9_ hydrochloride (Cayman) and TMAO-d_9_ (Toronto Research Chemicals) at 50 μM and 160 μM, respectively, in acetonitrile with 10 mM hydrochloric acid. The mixture was briefly vortexed immediately before adding 45 μL of ethyl bromoacetate (Sigma-Aldrich) solution (15 mg/mL, 1% [v/v] ammonium hydroxide in acetonitrile). The mixtures were incubated at room temperature for 30 minutes for derivatization, which was subsequently stopped by adding 185 μL of acetonitrile/water (95:5, v/v) solution. The mixtures were centrifuged at 18,000 *g* for 10 minutes at 4 °C, and 100 μL of the supernatant was transferred to a LC-MS glass vial and mixed with 50 μL of 1 M sodium heptanesulfonate solution (Sigma-Aldrich) in water.

Separation of TMAO, TMA, and labeled internal standards was obtained using a UPLC-MS/MS system (Waters) and a gradient separation combining an aqueous mobile phase A (0.1% formic acid in HPLC grade water, v/v) and organic mobile phase B (0.1% formic acid in acetonitrile, v/v), through a reversed phase high-strength silica T3 2.1 × 150 mm column (Waters) with a particle size of 1.8 μm. All solvents were HPLC grade (Fisher Scientific), and formic acid purity was 98%–100% (Merck). The starting separation conditions were 15% B and held for 1.5 minutes, increased to 55% B for 1.5 minutes, further increased to 99.9% B and held for 1 minute, before gradually returning to the initial conditions within 2 minutes. The total running time was 6 minutes at a flow rate of 0.35 mL/min. The injection volume was 2 μL, and the column temperature was maintained at 40 °C. The elution time for TMAO and the internal standard was 2.51 minutes and was 2.71 minutes for derivatized TMA and TMA-D_9_. The mass spectrometer conditions were performed at positive electrospray ionization mode. The capillary voltage was set at 1.5 kV. The desolvation temperature was 350 °C, the desolvation gas flow was 800 L/H, and the cone temperature was 150 °C. Multiple reaction monitoring ion pair masses for TMAO were 76→59 and 85→68 for TMAO-d_9_. Derivatized masses for TMA and TMA-d_9_ were 146→118 and 155→127, respectively.

Concentrations of TMAO and TMA in the samples were calculated based on the integrated peak area ratios obtained from MassLynx software (V2.1, Waters Corporation), which were proportional to the concentrations. A linear regression equation was constructed using Excel by plotting the calibration curve concentrations (ranging from 0.001 μM to 100 μM) for each compound on the x-axis and the peak area ratios for TMAO or TMA standards and their labeled counterparts on the y-axis. The concentrations of TMAO and TMA calculated were corrected for the corresponding sample dilution factor.

### Lymphocyte Isolation From Intestinal Tissues

Intestinal tissues were collected from 6 mice per group per sex for lymphocyte isolation. LPLs and IELs were isolated separately from the duodenum–jejunum, ileum, and colon. For IEL isolation, tissues were incubated in Hank’s Balanced Salt Solution (Sigma-Aldrich) containing 1 mM dithiothreitol (Sigma-Aldrich), 2% (v/v) fetal calf serum (FCS; Sigma-Aldrich), 100 U/mL penicillin (Sigma-Aldrich), and 100 μg/mL streptomycin (Sigma-Aldrich) for 15 minutes at 37 °C with rotation. Tissues were then transferred to Hank’s Balanced Salt Solution containing 1 mM EDTA (Sigma-Aldrich), 2% (v/v) FCS, penicillin, and streptomycin and incubated for a further 15 minutes at 37 °C. The resulting cell suspension was passed through a 100-μm cell strainer and collected on ice. The EDTA extraction was repeated once, and the 2 IEL fractions were pooled in a 50-mL Falcon tube.

Following IEL isolation, the remaining tissue left on the cell strainer was cut into small pieces and transferred into a well on a 6-well plate. A total of 7 mL of RPMI buffer (Thermo Fisher Scientific) containing 5% v/v FCS, 100 U/mL Collagenase VIII (Sigma-Aldrich), and 50 U/mL DNase (Sigma-Aldrich) was added into each well to isolate LPLs. The plate was placed on a shaker at low speed (100 rpm) for 30 minutes, and the solution containing tissue pieces in each well were transferred onto a 100-μm cell strainer to collect the flow-through containing LPLs in a 50-mL Falcon tube. The strainer and digestion vessel were rinsed with phosphate-buffered saline (PBS) containing 0.5% (v/v) bovine serum albumin and EDTA, and the resulting suspension was combined with the LPL fraction. The tubes containing IEL and LPL suspensions were centrifuged (10 minutes, 4° C, 500 *g*) and resuspended in 1 mL of PBS for staining.

Mesenteric lymph nodes (MLNs) were harvested and used as controls. MLNs were mechanically dissociated through a 100-μm cell strainer in RPMI medium supplemented with 5% (v/v) FCS. Cell suspensions were centrifuged at 500 × *g* for 5 minutes at 4 °C and resuspended in PBS for subsequent flow cytometric analysis.

### Flow Cytometry and Staining

A 24-color spectral flow cytometry panel was used to characterize intestinal immune cell populations. The panel included markers for T cells, B cells, NK cells, ILCs, macrophages, and myeloid-derived suppressor cells, together with functional and intracellular markers including interferon-γ, TNF-α, IL17, GrB, T-bet, RORγt, and Foxp3 (Table 2). Fluorescence-minus-one (FMO) controls were included for selected markers to facilitate consistent gating.

**Table 2:** Antibodies Used in Flow Cytometry

Antigen	Clone	Concentration, *μg/mL*	Fluorophore	Supplier
CD8	53-6.7	0.5	BUV395	BD Biosciences
CD4	GK1.5	0.5	BUV496	BD Biosciences
Ly6G	1A8	0.5	BUV661	BD Biosciences
CD3	145-2C11	1	BUV737	BD Biosciences
F4/80	BM8	0.5	Brilliant Violet 421	Biolegend
IFN-γ	XMG1.2	2	eFluor 450	eBioscience
CD11b	M1/70.15.11.5	n/a (dilution 1:800)	VioGreen	Miltenyi Biotec
Ly6C	HK1.4	0.025	Brilliant Violet 605	Biolegend
NK1.1	PK136	1	Brilliant Violet 650	Biolegend
T-bet	4B10	4	Brilliant Violet 711	Biolegend
B220	RA3-6B2	0.25	Brilliant Violet 785	Biolegend
CD200R	REA850	n/a (dilution 1:100)	Vio Bright B515	Miltenyi Biotec
CD127	A7R34	5	FITC	eBioscience
CD107a (LAMP-1)	1D4B	5	Biotin	Biolegend
CD206 (MMR)	C068C2	2	PerCP/Cyanine5.5	Biolegend
CD279 (PD-1)	J43	1	PerCP-eFluor 710	eBioscience
CD314 (NKG2D)	CX5	*2*	PE	eBioscience
RORγt	B2D	2	PE-eFluor 610	eBioscience
γδTCR	eBioGL3 (GL-3, GL3)	0.5	PE-Cyanine5	eBioscience
IL17	TC11-18H10.1	2	PE/Cyanine7	Biolegend
Foxp3	FJK-16s	1	APC	eBioscience
GrB	GB11	2	Alexa Fluor 647	BD Biosciences
CD45	30-F11	0.5	Alexa Fluor 700	Biolegend
TNF-α	MP6-XT22	2	APC/Cyanine7	Biolegend

Prior to staining, aliquots of IEL, LPL, and MLN cell suspensions were pooled to generate unstained and FMO controls. Cells were incubated with Fc receptor-blocking reagent (eBioscience) and stained with Zombie UV Fixable Viability Dye (BioLegend) for 20 minutes. Cells were subsequently stained with the extracellular antibody cocktail for 30 minutes and washed twice. For intracellular staining, cells were fixed and permeabilized using the Transcription Factor Fixation/Permeabilization Kit (eBioscience) for 30 minutes, followed by staining with intracellular antibodies for 30 minutes and washing.

Samples were acquired on a Cytek Aurora spectral flow cytometer using SpectroFlo software (Cytek Biosciences). Spectral unmixing was performed using appropriate single-stained reference controls, and FMO controls were used to define gating boundaries where required. Flow cytometry data were analyzed using FlowJo v10 (BD Biosciences). After auto-compensation during acquisition, compensation parameters were manually verified in FlowJo, and FMO controls were employed to refine gating.

### RAW264.7 Macrophage Culture and AlamarBlue Cytotoxicity Assay

Murine RAW264.7 macrophage (ATCC, LGC Prochem) were cultured in Dulbecco’s Modified Eagle Medium (Thermo Fisher Scientific) supplemented 10% v/v FCS (Thermo Fisher Scientific) at 37 °C in a humidified atmosphere with 5% CO_2_. Cells were passaged at approximately 80% confluence, and passages 5 to 8 were used for experiments. TMAO (99% purity, Sigma-Aldrich) and TMA 40% solution (Sigma-Aldrich) were dissolved in water at concentrations of 500 mM and 200 mM, respectively, as stock solutions were stored at −20 °C and diluted freshly with media prior to cell treatment.

Cytotoxicity was determined by incubating the cells with alamarBlue reagent (Invitrogen, Life Technologies) according to the manufacturer’s protocol. RAW264.7 cells were cultured at a density of 2 × 10^5^ cells per well in a 24-well plate overnight and treated with either TMAO at final concentrations of 5, 20, 100, and 500 μM or TMA at the final concentrations of 5, 20, 100, and 150 μM for 24 and 48 hours. Etoposide (Sigma-Aldrich) was used as a positive control at a final concentration of 125 nM. AlamarBlue was added at a final concentration of 10% for each well and incubated for 3 hours at 37 °C. Fluorescence was measured in a BMG FLUOstar Omega Plate Reader (BMG, Labtech) at excitation 560 nm/emission 590 nm. The cell viability of the treated cells was presented as a percentage of the negative control cells. Each sample was tested with technical triplicates, and 3 independent biological replicates were assessed.

### Measurement of Tumor Necrosis Factor-α in RAW264.7 Cell Culture Supernatants

RAW264.7 cells were treated with TMAO (50 μM) and TMA (150 μM) for 24 hours prior to LPS (*Escherichia coli* O111:B4) (10 ng/mL) treatment for 16 hours. The protein levels of TNF-α in the cell culture medium were determined by enzyme-linked immunosorbent assay kit against mouse TNF-α (R&D Systems; DY410) according to the manufacturer’s instructions. Each sample was tested with duplicates, and 3 independent biological replicates were assessed.

### HCT116 Cell Culture and Cytotoxicity Assay

Human colonic adenocarcinoma cell line HCT116 cells (ATCC, LGC Prochem) were cultured in RPMI 1640 medium (Thermo Fisher Scientific) with 10% FCS at 37 °C in a humidified atmosphere with 5% CO_2_. Cells at passages between 5 and 8 were used for experiments. For cytotoxicity measurement, HCT116 cells were seeded at a density of 3 × 10^4^ per well in a 24-well plate for overnight and treated with TMAO at final concentrations of 5, 20, 100, and 500 μM for 24 or 48 hours. Cell viability was assessed using the AlamarBlue assay as described above.

### Proliferation and Genotoxicity of HCT116 Cells Treated With Trimethylamine *N*-Oxide

The effects of TMAO on HCT116 cell proliferation and DNA damage were assessed by flow cytometric analysis of Ki-67 (Invitrogen) and γH2AX (BioLegend) staining, respectively, followed by flow cytometry analysis. HCT116 cells were cultured at a density of 4 × 10^5^ cells per well in a 6-well plate for overnight. Cells were treated with TMAO (50 μM) for 24 hours. Etoposide was used as a positive control at a final concentration of 125 nM and treated for 6 hours, as we previously observed a higher number of γH2AX-positive cells at this time point compared with 24 hours. The cells were collected using enzyme-free cell dissociation buffer (GIBCO), and subsequently fixed and permeabilized. The fixed cells were stained with Ki-67 and γH2AX antibodies and analyzed on a FACSymphony A3 flow cytometer (BD Bioscience). The data were analyzed using FlowJo v10.8 (BD Bioscience). Three independent biological replicates were performed.

### Statistical Analysis

All experiments in the cellular study were performed with 3 independent biological replicates. Data are expressed as the mean ± standard error of the mean. Group differences were assessed using 1-way analysis of variance followed by Tukey’s multiple comparisons test for comparisons among multiple groups. For circulation TMAO and TMA concentrations in patients, data normal distribution was tested using the Shapiro–Wilk test. Data with normal distribution was analyzed using the paired *t* test or the unpaired *t* test, and data that did not pass the normality test were analyzed using Wilcoxon matched pairs signed rank test. In mouse experiments, the data were analyzed using the either Kruskal–Wallis test or 1-way analysis of variance test. GraphPad Prism 9 software was used for statistical analysis. *P* < .05 was considered statistically significant.
